# Local Antibiotic Infusion in Periprosthetic Joint Infection Following Total Hip Arthroplasty

**DOI:** 10.3390/jcm13164848

**Published:** 2024-08-16

**Authors:** Atthakorn Jarusriwanna, Wenbo Mu, Javad Parvizi

**Affiliations:** 1 Rothman Orthopaedic Institute, Thomas Jefferson University, Philadelphia, PA 19107, USA; atthakorn.ton@gmail.com (A.J.); muwenbo8964@163.com (W.M.); 2 Department of Orthopaedics, Faculty of Medicine, Naresuan University, Phitsanulok 65000, Thailand; 3 Department of Orthopaedics, First Affiliated Hospital of Xinjiang Medical University, Ürümqi 830011, China; 4 Department of Surgical Sciences, Orthopedics and Traumatology, School of Medicine, Acibadem University, Istanbul 34752, Türkiye; 5 Sidney Kimmel Medical College, Thomas Jefferson University, Philadelphia, PA 19107, USA

**Keywords:** periprosthetic joint infection, hip arthroplasty, local antibiotic infusion, intra-articular antibiotic infusion

## Abstract

Local antibiotic infusion has emerged as a promising adjunctive therapy, delivering high concentrations of antibiotics directly to the infection site. This approach aims to enhance eradication of pathogens while minimizing systemic side effects associated with prolonged antibiotic use. This narrative review encompassed 10 articles focused on all three procedures of surgical intervention for periprosthetic joint injection (PJI) following total hip arthroplasty (THA): debridement, antibiotics, and implant retention (DAIR), single-stage revision arthroplasty, and two-stage revision arthroplasty. Recent studies report success rates ranging from 90 to 100% in patients undergoing DAIR, 82 to 100% in single-stage revision arthroplasty, and 80% in two-stage revision arthroplasty. The adjunctive use of local antibiotic infusion alongside surgical treatment for PJI following THA provides high success rates and is associated with low systemic complications, such as renal toxicity. Further research, particularly high-quality randomized controlled trials (RCTs), is warranted to validate and refine treatment protocols, ensuring consistent efficacy and safety.

## 1. Introduction

Periprosthetic joint infection (PJI) following total hip arthroplasty (THA) is a devastating complication that leads to significant morbidity and mortality. The management of this condition remains challenging due to various risk factors, which result in variable success rates in different specific surgical procedures. Factors such as patient comorbidities, the virulence of the infecting organisms, the duration of infection, and the timing of intervention all influence the outcomes of surgical treatments. The two-stage exchange THA has been reported to achieve infection eradication rates ranging from 77% to 89%, depending on the success criteria and various influencing factors [1,2,3]. The overall survival rate decreases over time, with 1-year, 3-year, and 5-year survival rates of 93%, 88%, and 80%, respectively. However, the 5-year overall mortality rate of 40.7% following this surgical procedure underscores the serious and often life-threatening nature of the disease and highlights the challenges associated with effectively managing and treating this complication [1]. Single-stage revision THA has recently gained popularity as it reduces the burden on patients by enhancing their quality of life and functional outcomes, allowing them to commence full rehabilitation immediately after surgery. Another advantage is the avoidance of morbidity and complications associated with multiple surgeries, a shorter total hospital stay, and reduced overall treatment costs [4]. A recent systematic review and meta-analysis revealed no significant difference in reinfection and reoperation rates compared to two-stage exchange arthroplasty [5]. Debridement, antibiotics, and implant retention (DAIR) is a valuable treatment option, offering a less invasive alternative to revision surgery, with success rates ranging from 58% to 83% [6]. Even with several surgical procedures to manage this condition, antimicrobials still play a major role in the treatment. Several factors are related to the success of the treatment for PJI, including the precise identification of the specific bacteria or microorganisms, antibiotic sensitivity, and the route and optimal duration of the antibiotic treatment [7].

Biofilm formation is a critical factor in the pathology of PJI. The bacteria in biofilms are embedded in a protective extracellular matrix, which shields them from the host’s immune response and antibiotic treatment, making infections particularly difficult to eradicate [8]. Additionally, biofilms evade the host’s immune system, as their dense structure prevents immune cells and antimicrobial agents from reaching the bacteria. This immune evasion, coupled with the difficulty of detecting biofilm-associated infections using traditional culture methods, complicates diagnosis and delays the initiation of appropriate treatment. The chronic nature of biofilm infections can lead to persistent and recurrent issues, necessitating more invasive and costly interventions, including complex surgical procedures and novel therapeutic strategies [9]. Furthermore, traditional interventions often exhibit inadequate responses to biofilm-associated infections, underscoring the need for innovative treatment approaches to effectively disrupt and eliminate biofilms. Local antibiotic delivery can deliver high concentrations of antibiotics directly to the infection site and enhance the efficacy of the treatment with the potential to penetrate the biofilm [10]. This review aimed to highlight the role and present the latest evidence on the efficacy of local antibiotic infusion in PJI following THA.

## 2. Materials and Methods

A comprehensive literature search was conducted using PubMed, EMBASE, and the Cochrane Central Register of Controlled Trials (CENTRAL). Briefly, free-text and thesaurus searches were performed to encompass the following three domains: (1) periprosthetic joint infection AND (2) total hip arthroplasty AND (3) local or intra-articular antibiotic infusion. The studies included in this review were full-text clinical studies, either retrospective cohorts, case–control studies, or RCTs, that focused on the adult population (age > 18 years) who underwent surgery for PJI following THA and received intra-articular antibiotic infusion postoperatively. Only studies written in English were considered, and there were no time restrictions. A narrative review was conducted based on the theoretical foundation of local antibiotic infusion in each treatment procedure for PJI following THA.

## 3. Theoretical Basis of the Local Antibiotic Infusion in PJI Management 

Local or intra-articular antibiotic infusion is a method of delivering high concentrations of antibiotics directly to the site of PJI. This approach involves administering antibiotics directly into the joint space through a catheter placed in the infected area. It aims to achieve therapeutic levels of antibiotics at the infection site while minimizing systemic exposure and potential side effects. The use of local antibiotic infusion in PJI management is considered in cases where systemic antibiotics alone may be insufficient to eradicate the infection, especially when dealing with biofilm-associated pathogens. By delivering antibiotics directly to the affected joint or tissue, local infusion can potentially enhance treatment efficacy by improving antibiotic penetration into the biofilm and targeting the infection more effectively [11]. In an animal model, Wei et al. [12] performed arthroplasty surgery in rats and inoculated the joints with methicillin-resistant *Staphylococcus aureus* (MRSA), followed by treatment with a vancomycin-cement spacer and a once-daily intra-articular injection of vancomycin (equivalent to 0.5 g of intra-articular vancomycin for a 70 kg patient) for 14 days. This treatment demonstrated a significant reduction in the bacterial colony count and less bone destruction compared to those in the rats that did not receive the intra-articular vancomycin injection. The liver and renal functions of all rats, evaluated by serum creatinine, aspartate aminotransferase (AST), and alanine transaminase (ALT), were within the normal range, and the serum vancomycin levels were sub-nephrotoxic.

For human studies, the direct administration of vancomycin into the joint has demonstrated high intrawound concentrations with low systemic levels. Specifically, the peak concentration of vancomycin in the joint typically occurs around 3 h after injection, with a half-life of approximately 7.2 h, and achieves subtherapeutic level after 64 h [13]. He et al. [14] found that the administration of intra-articular vancomycin 0.5 g once daily in patients with PJI has been shown to maintain high trough serum vancomycin concentrations in the synovial fluid before the next intra-articular dose, regardless of whether it is combined with intravenous (IV) administration. A systematic review by Bruyninckx et al. [15] includes PJI patients after total joint arthroplasty (TJA) who were treated with intra-articular antibiotic infusion for an average duration of 19 days. This study found an overall failure rate of approximately 11% and a complication rate of 18.5%, with most of the complications being non-catheter-related.

## 4. Adjuvant Local Antibiotic Infusion in Each Treatment Procedure for PJI Following THA

There are 10 studies included in the final review based on the treatment procedure for PJI following THA. Most of the literature focuses on single-stage revision (six studies), followed by two-stage revision (two studies), DAIR (one study), and a combination of single-stage and DAIR (one study). In studies that included all patients with PJI following THA, total knee arthroplasty (TKA), and other TJA, we extracted and reviewed data only for patients with PJI following THA. A summary of all studies is presented in Table 1.

## 5. Intra-Articular Infusion Technique

Local antibiotic infusion involves placing catheters directly into the joint cavity for delivery. While the Hickman catheter is well known for IV access, it could be adapted for this purpose. Gillard-Campbell et al. [17] provided an initiated subcutaneous tunnel by a laparoscopic Allis clamp and inserted the Hickman catheter through the fascia. The catheter was positioned through the skin in the anterior thigh and secured with a nylon suture. They started antibiotic infusion approximately 2 weeks after catheter insertion or when the insertion site was completely dry. During this interval, they flushed the catheter with heparin every 2 days until the start of antibiotic infusion. Additionally, they also heparinized the catheter after each administration of antibiotics to prevent occlusion. However, Whiteside and Roy [24] did not report the use of heparin to prevent occlusion of the catheter, and they started antibiotic infusion as soon as the incision was sealed and dry, which usually took 2 days. Aspiration and drainage were not performed to maintain the high concentration of antibiotics in the joint cavity. No complications related to the catheter were found after removal, such as a chronic fistula or significant drainage from the catheter site. This may be associated with tissue ingrowth around the catheter’s sealed fibrous cuff that was placed beneath the skin insertion. 

Another technique used to deliver intra-articular antibiotics is the three-branch (T-branch) catheter. Ji et al. [19,20], Li et al. [21,22], and Mu et al. [11] inserted a three-branch catheter into the hip joint cavity before closing the deep fascia, and the injection portal was placed proximally to the surgical wound. At the same time, a surgical suction drainage was placed distally to the hip joint. They started local antibiotic infusion immediately one day after surgery. The postoperative protocol included extracting synovial fluid for investigation, clamping the surgical drainage tube, and administration of intra-articular antibiotics. The distal drainage tube was then released varying from 6 to 20 h after local antibiotic infusion. Additionally, Ji et al. [19] performed repeated intra-articular injections and extractions of sterile saline in case the suction drainage was blocked. However, a fistula after catheter removal was observed, and the patient was treated with resuturing and compressive dressing [21,22].

Springer et al. [23] utilized an irrigation line connected to a temporary implantable spacer device designed to distribute local antibiotics into the intra-articular space and intramedullary canal (VT-X7), with an occlusive dressing connected to an external pump, through which the antibiotics are removed via a vacuum system.

## 6. Efficacy of DAIR Combined with Local Antibiotic Infusion

Mu et al. [11] conducted a retrospective study of 73 PJI patients who underwent DAIR for surgical treatment for PJI, with 32 of them being infected following THA. Their antibiotic protocol included IV pathogen-sensitive antibiotics for patients with a culture-positive infection and vancomycin 1 g for patients with a culture-negative infection for 14 days. In both groups, the intra-articular antimicrobial protocol comprised 0.5 g of intraoperative vancomycin powder, followed by pathogen-sensitive antibiotics if the culture was either multidrug-resistant bacteria, fungus, or a polymicrobial infection, whereas vancomycin 0.5 g in the morning and imipenem/cilastatin 0.5 g in the afternoon were given to patients with a culture-negative infection for 12–14 days. Oral antibiotics with quinolones and rifampicin were prescribed to patients in both groups for at least 1 month after IV and local antibiotic infusion. The results after an average follow-up duration of 63.8 months demonstrated a success rate of 90.63% without any reported systemic toxicity.

Another study on the efficacy of local antibiotic infusion after DAIR was conducted by Whiteside and Roy [24]. They included nine late-onset acute PJI patients who underwent DAIR and received intra-articular vancomycin infusions for 6 weeks. Of these, four patients had positive cultures for MRSA, and the rest of the patients had positive cultures for methicillin-sensitive *Staphylococcus aureus* (MSSA). They started vancomycin at a daily dosage of 100 mg in 3 mL of sterile water, and then the dose was increased to 400 or 500 mg in 5 or 6 mL of sterile water. The patients did not receive concomitant IV antibiotics during the intra-articular antibiotic infusions nor concomitant oral antibiotics after the discontinuation of the local antibiotic infusions. At a mean follow-up duration of 74 months, all patients remained free of infection without permanent damage to the kidney.

## 7. Efficacy of Single-Stage Revision Arthroplasty Combined with Local Antibiotic Infusion

A multicenter study by Antony et al. [16] demonstrated a 100% success rate with 4–6 weeks of intra-articular antibiotic infusion in patients who had previously experienced failure of PJI treatment (a recurrence or relapse of the infection). Their protocol included patients who had undergone revision surgery with prior administration of 4–6 weeks of IV or oral antibiotics and then experienced treatment failure according to clinical or laboratory evidence. They placed two Hickman catheters within the joint cavity and infused pathogen-sensitive antibiotics once or twice daily, most of which consisted of a combination of vancomycin and gentamicin. Concomitant systemic antibiotics were not used during the administration of the local antibiotic infusions. The results of this study found that no patients had positive cultures in their synovial fluid after the treatment. Whiteside and Roy [24] also conducted a retrospective study to evaluate the efficacy of local antibiotic infusion in 21 patients with chronic PJI following cemented THA and who underwent single-stage revision with cementless prosthesis. The local antibiotic infusion protocol for Gram-positive bacterial infection was similar to the protocol proposed in the same study involving late-onset acute PJI patients who underwent DAIR, where IV antibiotics were not administered to all patients. Additionally, a patient with a Gram-negative bacterial infection (*Serratia marcescens*) received a starting dose of 10 mg of gentamicin in 3 mL of normal saline, achieving a maintenance dose of 40 mg in 4 mL of normal saline once daily. In their report, there was one patient whose culture was positive for *Candida albicans* and who underwent re-revision surgery. This patient later received intra-articular, IV, and oral fluconazole for their treatment. The results of this study revealed that 95% of patients were free of infection at 1 year, and all remained free of signs and symptoms of infection at an average follow-up duration of 63 months. Nevertheless, there was report of patients experiencing elevated serum vancomycin levels or elevated serum blood urea nitrogen (BUN) and creatinine levels, which resolved after the discontinuation of intra-articular vancomycin. They later restarted local antibiotic infusions with a daily dose of 250 mg of vancomycin after renal function studies normalized for 1 week.

Ji et al. [18] conducted a retrospective study to report the outcome of cementless single-stage revision THA in patients with chronic PJI. They administered IV antibiotics to all patients for a mean of 14 days and employed once-daily local antimicrobial infusion protocols that included vancomycin alone, vancomycin and imipenem, fluconazole or voriconazole, and vancomycin and imipenem (or meropenem) for patients with a multidrug-resistant infection, culture-negative infection, fungal infection, and polymicrobial infection, respectively. From their protocol, the success rate in culture-negative-infection patients was 82.6%, without report of systemic toxicity. Another study by the same authors [19] evaluated the efficacy of local antimicrobial infusion in patients with culture-negative infections compared to those with culture-positive infections. In this study, only patients with a culture-positive infection with fungus or multidrug-resistant organisms received a daily intra-articular infusion of pathogen-sensitive antimicrobials, whereas a combination of 0.5 g of imipenem and 0.5 g of vancomycin was given to all patients with a culture-negative infection. Concomitant IV antibiotics were given for a mean of 14 days. At a mean follow-up duration of 53.2 months for culture-negative-infection patients, the success rate of revision THA was 89.5%, with only one patient experiencing a complication of renal insufficiency. No significant difference in the infection control rates was observed between culture-negative and culture-positive PJIs. Ji et al. [20] also reported the efficacy of local antibiotic infusion in patients with multiple failed surgical interventions for PJI. The local antimicrobial infusion protocol included 500 mg of vancomycin for Gram-positive bacteria, 0.5 g of meropenem or 1 g of imipenem for Gram-negative bacteria, and 0.1 g of voriconazole for fungal infection. They also administered concomitant IV antibiotics for a mean of 17 days. Of the 29 hip PJI patients, 26 patients (89.7%) were successful without relapse of the infection at a 7-year follow-up.

A retrospective study by Li et al. [21] revealed the outcome of local antimicrobial infusion in chronic PJI patients with a polymicrobial infection. Patients with a combination of Gram-positive and Gram-negative bacterial infections received a daily dose of 0.5 g of vancomycin and 0.5 g of imipenem, while patients with a combination of Gram-positive bacterial and fungal infections received a daily dose of 0.5 g of vancomycin and 0.2 g of fluconazole. Concomitant IV antibiotics were administered to all patients. However, this study reported the overall failure cases without specifying whether they were hip or knee PJI patients, within a mean follow-up period of 41 months. The secondary outcome demonstrated no radiographic migration of the implants in patients with revision THA, and one patient had a radiolucent line at the proximal stem, but the diameter was <2 mm with no progression. Li et al. [22] also conducted another retrospective study including 22 chronic hip PJI patients who underwent single-stage revision combined with intra-articular antibiotic infusion. They provided intraoperative intra-articular carbapenems (0.5 g of imipenem or meropenem) and once-daily intra-articular carbapenems (same dosage) for Gram-negative-bacteria PJI patients. For polymicrobial infections, they administered a combination of 0.5 g of vancomycin and 0.5 g of carbapenems (imipenem or meropenem) both intraoperatively and once daily postoperatively. As with the previous study, they did not specify whether the failure cases were patients with hip or knee PJI (except for one patient who experienced failure after revision TKA), but no patients developed renal failure.

## 8. Efficacy of Two-Stage Revision Arthroplasty Combined with Local Antibiotic Infusion

Two-stage revision arthroplasty with adjuvant local antibiotic infusion was presented in two studies. Gillard-Campbell et al. [17] reported on 12 PJI patients following THA with an average follow-up period of 2.9 years. Of those 12 cases, only 5 chronic PJI patients underwent two-stage revision, whereas 7 acute PJI cases were treated with catheterization and intra-articular antibiotic infusion only as an alternative to incisional and drainage (I&D). The local antibiotic protocol included the combination of 500 mg of vancomycin in 5 mL of saline and 2 g of cefazolin in 5 mL of saline daily. Nevertheless, they monitored the serum vancomycin level weekly, and the dosage of the antibiotics was adjusted based on the random vancomycin trough. Systemic antibiotics were not used during the administration of local antibiotic infusion, and the two-stage revision was then performed after 6 weeks of local antibiotic infusion. In this series, only one patient with chronic PJI who was treated with two-stage revision combined with local antibiotic infusion experienced treatment failure and required re-revision surgery (an 80% success rate). 

Springer et al. [23] conducted an RCT to compare the safety profiles between a novel protocol of cyclical, intra-articular antibiotic irrigation and a standard protocol of two-stage exchange arthroplasty. Fourteen hip PJI patients were allocated to the experimental group, and twelve hip PJI patients were allocated to the control group. The experimental group received a novel spacer device (VT-X7) as mentioned earlier during the interstage period, with an intra-articular irrigation of 80 mg of tobramycin in 50 mL of saline daily (a 2 h soak and a 30 min vacuum), combined with cyclical vancomycin irrigation over the rest of the day (125 mg of vancomycin in 50 mL of saline, with a 30 min soak and a 30 min vacuum per cycle) for 7 days. After the two-stage revision, all patients received systemic antibiotics for 12 weeks. Their protocol reported that the concentrations of both vancomycin and tobramycin were below the established systemic toxicity levels, with no significant difference in the number of patients experiencing adverse events compared to that of the control group. Nevertheless, this study did not report the surgical survivorship in terms of surgery failure or re-revision rates.

## 9. Future Directions

Due to the various protocols and heterogeneity in types and durations of adjunctive local antibiotic infusion for PJI following THA, coupled with the lack of control groups and small cohort sizes in recent studies, the routine administration of intra-articular antibiotics in the treatment of PJI is not yet justified [25]. The future of this approach holds significant potential, promising to address current challenges and improve clinical outcomes. Personalized medicine will play a critical role, with the development of tailored antibiotic regimens based on the precise identification of pathogens and individual patient factors such as immune status and comorbidities. The exploration of combination therapies, where multiple antibiotics work synergistically from systemic and local sources, holds promise for further enhancing treatment effectiveness. Comparative studies with long-term follow-up are essential to generate robust evidence on the efficacy, safety, and cost-effectiveness of this approach, guiding the development of standardized guidelines and protocols. Interdisciplinary collaboration will be crucial in addressing the complex challenges of PJI, paving the way for improved patient outcomes and reduced infection recurrence rates.

## 10. Conclusions

Local antibiotic infusion in PJI following THA highlights its efficacy in effectively managing and treating PJIs, especially when used alongside surgical interventions. It has shown success rates ranging from 90% to 100% in patients undergoing DAIR and 82% to 100% in those undergoing single-stage revision arthroplasty. For patients undergoing two-stage revision arthroplasty, the success rate has been reported to be 80%. This technique minimizes systemic exposure to antibiotics, thereby reducing the risk of systemic toxicity, including renal failure and other adverse effects. While not a standalone treatment, local antibiotic infusion is a valuable adjunct to surgical procedures, enhancing the likelihood of successful outcomes and implant retention. Further high-quality RCTs are needed to solidify these findings and optimize treatment protocols.

## Figures and Tables

**Table 1:** A summary of the studies included in this review.

Authors (Year)	Country	PJI Location	Surgical Treatment Procedure	No. of Patients *	Organisms	Local Infusion Protocol	Catheter Used for Local Infusion	Duration of Local Infusion
Antony et al. (2015) [16]	USA	Hip, knee, elbow	Single-stage	20	Gram-positive bacteria, Gram-negative bacteria, culture-negative infection, fungus, multidrug-resistant organisms	- Pathogen-sensitive antibiotics (culture-positive infection) - Empirically selected antibiotics based on the most likely pathogens (culture-negative infection)	Hickman catheter	4–6 weeks
Gillard-Campbell et al. (2021) [17]	USA	Hip, knee	Two-stage	5	Not reported	Vancomycin and cefazolin	Hickman catheter	6 weeks
			Catheterization infusion	7				
Ji et al. (2019) [18]	China	Hip	Single-stage	50	Culture-negative infection, fungus, multidrug-resistant and polymicrobial organisms	- Vancomycin and imipenem (culture-negative infection) - Vancomycin (multidrug-resistant organisms) - Fluconazole or voriconazole (fungus) - Vancomycin and carbapenems (polymicrobial organisms)	Not reported	- Mean 16 days (range 12–20 days) for culture-negative infection - Mean 18 days (range 12–35 days) for fungus and multidrug-resistant organisms
Ji et al. (2020) [19]	China	Hip, knee	Single-stage	99	Culture-negative infection, fungus, multidrug-resistant organisms	- Vancomycin and imipenem (culture-negative infection) - Pathogen-sensitive antibiotics (fungus and multidrug-resistant organisms)	Three-branch catheter	- Mean 16 days (range 12–20 days) for culture-negative infection - Mean 18 days (range 12–35 days) for fungus and multidrug-resistant organisms
Ji et al. (2022) [20]	China	Hip, knee	Single-stage	29	Gram-positive bacteria, Gram-negative bacteria, fungus, culture-negative infection, multidrug-resistant and polymicrobial organisms	- Vancomycin (Gram-positive bacteria) - Carbapenems (Gram-negative bacteria) - Voriconazole (fungus) - Vancomycin and carbapenems (culture-negative infection) - Other combinations for polymicrobial organisms	Three-branch catheter	Mean 16 days (range 12–21 days)
Li et al. (2022) [21]	China	Hip, knee	Single-stage	69	Gram-positive bacteria, Gram-negative bacteria, fungus, multidrug-resistant and polymicrobial organisms	- Vancomycin (Gram-positive bacteria) - - Imipenem (Gram-negative bacteria) - Vancomycin and imipenem (Gram-positive and negative bacteria) - Vancomycin and fluconazole (Gram-positive bacteria and fungus)	Three-branch catheter	- Mean 15 days (range 13–28 days) for monomicrobial infection - Mean 16 days (range 14–29 days) for polymicrobial infection
Li et al. (2023) [22]	China	Hip, knee	Single-stage	22	Gram-negative bacteria and polymicrobial organisms	- Carbapenems (Gram-negative bacteria) - Vancomycin and carbapenems (polymicrobial organisms)	Three-branch catheter	- Mean 14 days (range 13–17 days) for monomicrobial infection - Mean 15 days (range 14–19 days) for polymicrobial infection
Mu et al. (2020) [11]	China	Hip, knee	DAIR	32	Gram-positive bacteria, Gram-negative bacteria, fungus, culture-negative infection, multidrug-resistant and polymicrobial organisms	- Pathogen-sensitive antibiotics (culture-positive infection) - Vancomycin and imipenem/cilastatin (culture-negative infection)	Three-branch catheter	- 14 days for culture-positive infection - 12–14 days for culture-negative infection
Springer et al. (2024) [23]	USA	Hip, knee	Two-stage	14	Not reported	Tobramycin and vancomycin	Irrigation line connected to a short-term implantable spacer (VT-X7)	7 days
Whiteside and Roy (2017) [24]	USA	Hip	Single-stage	21	Gram-positive bacteria, Gram-negative bacteria, fungus, multidrug-resistant organisms	- Vancomycin (Gram-positive bacteria) - Gentamicin (Gram-negative bacteria) - Fluconazole (fungus)	Hickman catheter	6 weeks
			DAIR	9				

* Only patients with PJI following THA and who received local antimicrobial infusion.

## Data Availability

No new data were created or analyzed in this study. Data sharing is not applicable to this article.

## References

[1] Kildow B.J., Springer B.D., Brown T.S., Lyden E., Fehring T.K., Garvin K.L. (2022). Long Term Results of Two-Stage Revision for Chronic Periprosthetic Hip Infection: A Multicenter Study. J. Clin. Med..

[2] Goumenos S., Hardt S., Kontogeorgakos V., Trampuz A., Perka C., Meller S. (2024). Success Rate After 2-Stage Spacer-Free Total Hip Arthroplasty Exchange and Risk Factors for Reinfection: A Prospective Cohort Study of 187 Patients. J. Arthroplast..

[3] Akgün D., Müller M., Perka C., Winkler T. (2019). High cure rate of periprosthetic hip joint infection with multidisciplinary team approach using standardized two-stage exchange. J. Orthop. Surg. Res..

[4] Bialecki J., Bucsi L., Fernando N., Foguet P., Guo S., Haddad F., Hansen E., Janvari K., Jones S., Keogh P. (2019). Hip and Knee Section, Treatment, One Stage Exchange: Proceedings of International Consensus on Orthopedic Infections. J. Arthroplast..

[5] Zhao Y., Fan S., Wang Z., Yan X., Luo H. (2024). Systematic review and meta-analysis of single-stage vs two-stage revision for periprosthetic joint infection: A call for a prospective randomized trial. BMC Musculoskelet. Disord..

[6] Scheper H., Gerritsen L.M., Pijls B.G., Van Asten S.A., Visser L.G., De Boer M.G.J. (2021). Outcome of Debridement, Antibiotics, and Implant Retention for Staphylococcal Hip and Knee Prosthetic Joint Infections, Focused on Rifampicin Use: A Systematic Review and Meta-Analysis. Open Forum Infect. Dis..

[7] Le Vavasseur B., Zeller V. (2022). Antibiotic Therapy for Prosthetic Joint Infections: An Overview. Antibiotics.

[8] Staats A., Li D., Sullivan A.C., Stoodley P. (2021). Biofilm formation in periprosthetic joint infections. Ann. Jt..

[9] González J.F., Hahn M.M., Gunn J.S. (2018). Chronic biofilm-based infections: Skewing of the immune response. Pathog. Dis..

[10] Steadman W., Chapman P.R., Schuetz M., Schmutz B., Trampuz A., Tetsworth K. (2023). Local Antibiotic Delivery Options in Prosthetic Joint Infection. Antibiotics.

[11] Mu W., Xu B., Guo W., Ji B., Wahafu T., Cao L. (2021). Outcome of Irrigation and Debridement with Topical Antibiotics Delivery for the Management of Periprosthetic Joint Infection Occurring within 3 Months Since the Primary Total Joint Arthroplasty. J. Arthroplast..

[12] Wei J., Tong K., Wang H., Wen Y., Chen L. (2022). Intra-articular versus systemic vancomycin for the treatment of periprosthetic joint infection after debridement and spacer implantation in a rat model. Bone Jt. Res..

[13] Johnson J.D., Nessler J.M., Horazdovsky R.D., Vang S., Thomas A.J., Marston S.B. (2017). Serum and Wound Vancomycin Levels after Intrawound Administration in Primary Total Joint Arthroplasty. J. Arthroplast..

[14] He J.W., Wang J., Cao L., Zhang X.G., Li G.Q., Xu B.Y., Ji B.C., Ge S.Y., Yang J.H. (2021). Serum and Synovial Vancomycin Concentrations in Patients with Prosthetic Joint Infection after Intra-articular Infusion. Eur. J. Drug Metab. Pharmacokinet..

[15] Bruyninckx S., Metsemakers W.J., Depypere M., Henckaerts L., Van Den Hout E., Onsea J., Ghijselings S., Vles G.F. (2024). Local antibiotic delivery via intra-articular catheter infusion for the treatment of periprosthetic joint infection: A systematic review. Arch. Orthop. Trauma Surg..

[16] Antony S.J., Westbrook R.S., Jackson J.S., Heydemann J.S., Nelson J.L. (2015). Efficacy of Single-stage Revision with Aggressive Debridement Using Intra-articular Antibiotics in the Treatment of Infected Joint Prosthesis. Infect. Dis..

[17] Gaillard-Campbell D., Gross T.P., Webb L. (2021). Antibiotic Delivery via Hickman Catheter for the Treatment of Prosthetic Joint Infection. Orthopedics.

[18] Ji B., Wahafu T., Li G., Zhang X., Wang Y., Momin M., Cao L. (2019). Single-stage treatment of chronically infected total hip arthroplasty with cementless reconstruction: Results in 126 patients with broad inclusion criteria. Bone Jt. J..

[19] Ji B., Li G., Zhang X., Wang Y., Mu W., Cao L. (2020). Effective treatment of single-stage revision using intra-articular antibiotic infusion for culture-negative prosthetic joint infection. Bone Jt. J..

[20] Ji B., Li G., Zhang X., Xu B., Wang Y., Chen Y., Cao L. (2022). Effective single-stage revision using intra-articular antibiotic infusion after multiple failed surgery for periprosthetic joint infection: A mean seven years’ follow-up. Bone Jt. J..

[21] Li Y., Zhang X., Guo X., Wulamu W., Yushan N., Ji B., Cao L. (2022). Effective Treatment of Single-Stage Revision Using Intra-Articular Antibiotic Infusion for Polymicrobial Periprosthetic Joint Infection. J. Arthroplast..

[22] Li Y., Zhang X., Ji B., Wulamu W., Yushan N., Guo X., Cao L. (2023). One-stage revision using intra-articular carbapenem infusion effectively treats chronic periprosthetic joint infection caused by Gram-negative organisms. Bone Jt. J..

[23] Springer B.D., Higuera-Rueda C.A., De Beaubien B.C., Warner K.D., Glassman A.H., Parvataneni H.K., Piuzzi N.S. (2024). Safety Profile of Seven-Day Intra-articular Antibiotic Irrigation for the Treatment of Chronic Periprosthetic Joint Infection: A Prospective Randomized Phase II Comparative Study. J. Arthroplast..

[24] Whiteside L.A., Roy M.E. (2017). One-stage Revision with Catheter Infusion of Intraarticular Antibiotics Successfully Treats Infected THA. Clin. Orthop. Relat. Res..

[25] Whiteside L.A., Beaubien B., Martin K.E., Ferry C., Parvizi J., Gehrke T. (2018). Is there a role for direct intra-articular antibiotic infusion following irrigation and debridement for PJI?. Proceedings of the Second International Consensus Meeting on Musculoskeletal Infection.

